# Gender Influence on Students, Parents, and Teachers’ Perceptions of What Children and Adolescents in Germany Need to Cycle to School: A Concept Mapping Study

**DOI:** 10.3390/ijerph17186872

**Published:** 2020-09-20

**Authors:** Dorothea M. I. Schönbach, Catherina Vondung, Lisan M. Hidding, Teatske M. Altenburg, Mai J. M. Chinapaw, Yolanda Demetriou

**Affiliations:** 1 Department of Sport and Health Sciences, Technical University of Munich, 80992 Munich, Germany; yolanda.demetriou@tum.de; 2 Department of Natural and Sociological Sciences, Heidelberg University of Education, 69120 Heidelberg, Germany; vondung@ph-heidelberg.de; 3 Department of Public and Occupational Health, Amsterdam Public Health Research Institute, Amsterdam UMC, Vrije Universiteit Amsterdam, 1081 BT Amsterdam, The Netherlands; l.hidding@amsterdamumc.nl (L.M.H.); t.altenburg@amsterdamumc.nl (T.M.A.); m.chinapaw@amsterdamumc.nl (M.J.M.C.)

**Keywords:** childhood, adolescence, sex, active commuting to school, bicycle

## Abstract

Active commuting to school is highly recommended for several reasons, and in the decision-making process for doing so, a child interacts with parents and teachers. Until now, these three interactors’ gender-specific perspectives on children and adolescents’ need for cycling to school have been unavailable. Thus, our concept mapping study analyzed the needs of 12- to 15-year-olds in Germany for cycling to and from school daily, as perceived by students, parents, and teachers stratified by gender. From November 2019 to February 2020, 136 students, 58 parents, and 29 teachers participated. Although 87.8% of girls and 100% of boys owned a bicycle, only 44.4% of girls and 72.9% of boys cycled to school. On average, girls cycled to school on 1.6 ± 2.0 days a week and boys on 2.7 ± 2.0 days a week. A “bicycle and related equipment,” the “way to school,” and “personal factors” were reported needs, perceived by students and teachers of both genders and by mothers. Girls reported the additional gender-specific need for “social behavior in road traffic,” mothers and female teachers reported “role of parents,” and female teachers reported a “sense of safety.” This study’s findings could inspire the development of school-based bicycle interventions.

## 1. Introduction

Only 26% of children and adolescents aged 3 to 17 in Germany achieve the physical activity (PA) as described by the guidelines of the World Health Organization (WHO) [1]. Active commuting to school (ACTS) is regarded as an additional opportunity to increase PA before and after school and is highly recommended for school-aged children and adolescents [2]. Cycling is one mode of ACTS and has additional benefits compared to walking for the following reasons: (i) Compared to 25% of walkers, 36% of children and adolescents who cycle to school meet the weekly PA recommendation [3]; (ii) ACTS by bicycle has been generally associated with higher PA intensity than walking, with positive effects on cardiovascular fitness in children and adolescents [4] leading to a risk reduction of developing cardiovascular diseases; (iii) cycling increases the mobility of students who need to manage a longer home-to-school distance when engaging in ACTS [5,6]; (iv) in cities, a bicycle is considered the fastest means of transportation for distances less than 5 km [7], which is more time-efficient (especially when car traffic is congested); and v) ACTS by bicycle is more positively associated with cycling than walking to other destinations [3], possibly establishing a potentially lifelong active travel (AT) routine by bicycle. Over time, this maintained behavior routine is useful because it predicts PA in adults [8]. Despite these well-known benefits of ACTS by bicycle and the fact that 57% to 98% of children and adolescents aged up to 17 years in Germany own a bicycle [9], why only 8% [10] to 22.2% [11] of them cycle to and from school remains as yet an unknown. Furthermore, the reasons why more boys (23.8%) than girls (20.6%) in Germany cycle to school are still unclear [11]. In a recent systematic review of school-based ACTS interventions focusing on cycling, we found that only one in seven strategies was promising and that two grade levels between 3rd and 7th grade were chosen [12]. Moreover, analysis of gender differences has been performed for only one intervention, which indicated an unexplained beneficial effect on boys but not on girls [13].

According to the Model of Children’s Active Travel (M-CAT) [14], the main factors influencing children’s travel behavior include “objective characteristics” of the child (e.g., age, gender, school attended), parent (e.g., social status) or family (e.g., size), and further objective elements in physical (e.g., population density), economic (e.g., costs), or political-socio-cultural environments (e.g., school). Some previous research indicates that parents’ gender predicts positive associations between parental characteristics and a child’s ACTS (e.g., employed mothers [15], mothers actively commuting to work [16]). Moreover, M-CAT considers parent and child’s “perception elements” including attitudes (e.g., benefits or risks), the environment (e.g., favorable or unfavorable), and the child (e.g., sense of responsibility, knowledge of road safety, cycling skills) [14]. Because perception is based on “objective characteristics” [14], it can be influenced by the child or parents’ gender, so perception can also impact on the child’s ACTS. Previous research has reported influential factors identified by both parents of children aged 9 to 12 (e.g., perceived convenience of using the car to drive the child to school), whereas other factors were gender-specific to mothers (e.g., a child’s lack of interest) [17]. In conclusion, interaction among all these factors influences the outcome, i.e., parents and children’s decisions on children’s engagement in ACTS, as well as events occurring during children’s engagement (e.g., bullying) [14]. According to M-CAT, children make the final decision on whether they engage in a certain behavior [14], making them experts on their own behavior [18]. Their autonomy, independence, and personal responsibility increase with maturation, while the parents’ role and influence as supporters or decision-makers (i.e., ultimate allowance or restriction) simultaneously decrease [14]. Besides the child and its parents, M-CAT mentions schoolteachers as important interactors in the socialization process of ACTS [14]. In addition to the teaching mission, schoolteachers also follow an educational mission according to German laws and are commonly seen as role models on who should practice whatever they emphasize in school lessons [19]. Contrary to previous research on parents, we could not find a gender-specific analysis of teachers’ perspectives on ACTS.

Following this, parents at home and teachers at school educate and observe the child, making them experts on the child’s behavior and needs [20]. Parents and teachers can be aware of aspects influencing their decision to support the child’s ACTS [14], but the child, due to strengths, deficits, and stage of maturation, might not perceive them. This circumstance has already been confirmed in previous research, in which parents and their child had different perspectives on barriers of ACTS [15] and, conversely, needs. In addition, parents identified more barriers to ACTS than children [15]. Moreover, complementary and stimulating impulses of the child, parents, and teachers’ perceptions, especially in the gender context [14], might favor a successful socialization process of ACTS [21].

Therefore, our concept mapping study analyzed how perceptions of students, parents, and teachers differed by gender about what children and adolescents aged 12 to 15 in Germany need to cycle daily to and from school. Knowledge of potential similarities and discrepancies in gender-specific perspectives of students, parents, and teachers on perceived needs is necessary to develop future gender-sensitive, school-based bicycle interventions.

## 2. Materials and Methods

This study used the concept mapping method, a mixed method that combines quantitative and qualitative research [22,23]. It follows a participatory approach based on group processes [22,23] and generally consists of six steps [24]. First, the study is prepared by defining the participants and developing a main question. The second step includes the generation of participants’ answers to the main question. Third, participants cluster all unique answers from step two into groups of similar content and rate these answers on importance and feasibility. The fourth step includes the use of a computer program for a (hierarchical) cluster analysis and multidimensional scaling (multivariate statistical analyses). These analyses result in the representation of unique answers, obtained in the second step and structured in the third step, arranged as dots on a two-dimensional concept map. Distances between dots provide information about the frequency with which participants clustered unique answers in the same groups of similar content (i.e., the closer, the more often; the wider, the rarer). In step five, researchers interpret the concept map by identifying an adequate number of relevant clusters and, subsequently, label clusters according to their content based on participants’ suggestions. The sixth step includes use of the concept map to plan or evaluate further research.

### 2.1. Recruitment of Participants

To address ACTS needs in both urban and suburban living areas [6,11,25] and to address the age group (older than 12 years) in which cycling-to-school rates are low [26], in October 2019, an invitation letter was sent to four secondary schools in urban or suburban areas in Germany. Three schools (one urban; two suburban), each including two classes of 7th and/or 8th graders aged 12 to 15, agreed to participate in the study. Parents and teachers were also invited to participate. Prior to the study’s beginning, parents and teachers received an information letter, but only children whose parents, parents and teachers who provided signed consent forms participated. To ensure anonymity and to connect each individual’s data throughout all concept mapping sessions, participants were instructed to create a five-digit ID code themselves. In all, 136 students, 58 parents, and 29 teachers participated in at least one of the sessions (drop-out rates: 26.5% for students; 79.3% for parents; 62.1% for teachers).

### 2.2. Concept Mapping Sessions

#### 2.2.1. Students

All concept mapping sessions for students were conducted face-to-face at schools and were supervised by at least one trained researcher (D.M.I.S./C.V.). In each class, the sessions occurred during two regular lessons, i.e., 90 min. Based on schools’ availability of sufficient computers and/or stable internet connections, sessions were conducted using either a printed or an online version. Independent of both media, sessions followed exactly the same procedure.

The first concept mapping sessions with students took place in November and December 2019. At three schools, 123 students (49 females, 72 males, 2 diverse), aged 13.1 ± 0.9 years, from six classes (22 to 32 students per class) participated. During the first session, students completed a printed or an online questionnaire via the program Survalyzer [27]. This questionnaire was structured in three sections (see Table A1): (1) personal characteristics, e.g., age and gender (see Table A2), (2) a warm-up question (why do or don’t you cycle to school?), and (3) the main question (what do you need to cycle to and from school on a daily basis?). The warm-up question served as an icebreaker to introduce “cycling to school” to the students. To answer the main question, students had an individual and a group brainstorming phase. During individual brainstorming, students were stimulated to list as many answers to the main question as they could. During group brainstorming, individual students shared their written answers and checked their clarity, resulting in a list of unique answers for each class. After all classes had completed the first session, D.M.I.S. created a single list that included all unique answers from all six classes. The working process of D.M.I.S. was checked by the second researcher S.M., and any discrepancies were resolved through discussion. As a result, D.M.I.S. entered a list of 98 unique answers into the rating and clustering program Ariadne [28].

The second concept mapping sessions took place in January and February 2020; they were completed by 100 students (35 females, 64 males, 1 diverse). Here, students were asked to rate all 98 listed answers (paper/pencil with answers printed in a table or online via the program Ariadne) for both (a) importance and (b) feasibility on a five-point Likert scale (1 = very unimportant/unfeasible, 2 = unimportant/unfeasible, 3 = neutral, 4 = important/feasible, 5 = very important/feasible). Ninety-three (34 females, 59 males) and 83 students (32 females, 50 males, 1 diverse) completed the importance and feasibility ratings, respectively. Furthermore, students were asked to cluster all 98 answers (paper/pencil with answers printed on cards or online via the program Ariadne) in two to ten self-titled topic groups based on similarities between answers, with at least two answers in each; a “miscellaneous” pile was not allowed. Based on their individual ID code, each student used a personal link to access the tasks in Ariadne. Eighty-four students (30 females, 53 males, 1 diverse) completed the clustering task. Results from students who worked on the paper/pencil version were entered into Ariadne by D.M.I.S.

#### 2.2.2. Parents and Teachers

All concept mapping sessions for parents and teachers were conducted online at home, without researchers’ supervision. Prior to each session, parents and teachers received an information letter. During each working period per session, parents and teachers received a reminder asking them to participate in the study, in case they had not done so already.

The first concept mapping session with parents and teachers took place in November and December 2019. Participants included 42 parents (34 females, 8 males) aged 47.8 ± 5.5 years and 27 teachers (14 females, 13 males) aged 39.4 ± 10.9 years. During the first concept mapping session, parents and teachers completed separate online questionnaires via the program Survalyzer. Each questionnaire was structured in three sections (see Table A3 and Table A4): (1) personal characteristics, e.g., age and gender (see Table A5 and Table A6), (2) a warm-up question (parents: why does or doesn’t your child cycle to school?; teachers: why do or don’t your students cycle to school?), and (3) the main question (parents: what does your child need to cycle to and from school daily?; teachers: what do your students need to cycle to and from school daily?). The warm-up question served as an icebreaker to introduce “cycling to school” to parents and teachers. For the main question, parents and teachers listed as many answers as they could. After the first session, D.M.I.S. created lists that included all unique answers provided by parents and teachers, respectively. The working process of D.M.I.S. was checked by a second researcher (parents: P.W.; teachers: L.D.), and any discrepancies were resolved through discussion. As a result, P.W./L.D. (checked by D.M.I.S. and C.V.) entered parents’ 90 and teachers’ 94 unique answers into Survalyzer.

Because they completed the online questionnaire at home, parents and teachers could not participate in group brainstorming on the main question. Thus, in January 2020, an additional session was conducted in which 29 parents (10 females, 4 males, 15 unknown) and 7 teachers (1 female, 4 males, 2 unknown) checked the clarity of answers to the main question. Furthermore, parents and teachers could add new answers if inspired by other participants’ answers. After the second session, D.M.I.S. revised and combined their answers where necessary, based on parents/teachers’ comments, and created final lists that included all unique answers provided by parents and teachers, respectively. The working process of D.M.I.S. was checked by a second researcher (parents: P.W.; teachers: L.D.), and any discrepancies were resolved through discussion. As a result, D.M.I.S. entered revised lists of 90 parents’ answers and 94 teachers’ answers into Ariadne.

The third concept mapping session took place in February 2020, and was completed by 12 parents (9 females, 2 males, 1 unknown) and 11 teachers (6 females, 5 males). Here, parents and teachers rated all 90 and 94 answers listed online, via Ariadne, for both (a) importance and (b) feasibility on a five-point Likert scale (1 = very unimportant/unfeasible, 2 = unimportant/unfeasible, 3 = neutral, 4 = important/feasible, 5 = very important/feasible). Twelve parents (9 females, 2 males, 1 unknown) and 10 teachers (5 females, 5 males) completed the importance and feasibility rating. Furthermore, parents and teachers were asked to cluster all 90 and 94 answers listed online, via Ariadne, in two to ten self-titled topic groups based on similarities between answers with at least two answers in each; a “miscellaneous” pile was not allowed. Based on their individual ID code, each parent and teacher used a personal link to access the tasks in Ariadne. The clustering task was completed by 11 parents (9 females, 1 male, 1 unknown) and 10 teachers (5 females, 5 males).

### 2.3. Statistical Analyses and Interpretation of Concept Maps

Descriptive data from students, parents, and teachers as well as these groups’ statistical gender differences (female vs. male) were analyzed using the program IBM SPSS Statistics 25 [29]. Additionally, this program was used to determine the within and between variance of days per week students cycled to the three participating schools by calculating an intraclass correlation coefficient (ICC) [30]. In the development of future intervention designs, the ICC is relevant for dealing with potential variance among participating schools.

Only students, parents, and teachers who reported their gender as female or male, and completed at least one of the two rating tasks (importance or feasibility) or the clustering task were included in analyses. The small number of fathers completing rating tasks (*n* = 2) and the clustering task (*n* = 1) did not allow for separate data analysis. Each subgroup rated and clustered the same answers, and only the analysis was stratified by gender using Ariadne. D.M.I.S. looked at all possible options of clusters, illustrated in a hierarchical cluster tree, to define an adequate number of relevant clusters for the concept map. A hierarchical cluster tree arranges all answers in one cluster at first and suggests how this cluster can be further split into two, three, four, or more clusters based on how students, mothers, or teachers clustered the answers. When considered necessary, items were reallocated into already existing clusters (indicated by arrows) or newly created clusters (indicated by circles) to ensure plausibility of answers in clusters. These procedures were checked by a second researcher (students: S.M.; parents: P.W.; teachers: L.D.). Any discrepancies were resolved through discussion. Lastly, all clusters were named according to suggestions by students, mothers, and teachers. For each cluster, average ratings of both importance and feasibility were calculated and descriptively described. These average ratings were based on the mean individual rating of all answers in each cluster. As Ariadne did not provide the participants’ individual rating of all answers in each cluster, the appropriate statistical test for ordinal data (U-test) was not applicable to analyze differences in ratings [31].

## 3. Results

### 3.1. Cycling Behavior in Students

In total, 87.8% of girls and 100% of boys owned a bicycle (see Table A2). However, 44.4% of girls and 72.9% of boys cycled to school, but of these, 68.4% of girls and 62.7% of boys did not cycle to school daily. Girls cycled to school on 1.6 ± 2.0 days a week and boys on 2.7 ± 2.0 days a week.

Within and between the three participating schools, the variance of days per week students cycled to school, calculated with an ICC for 114 valid girls and boys, was 0.2 (high school in an urban area with 7th graders: 40 students; junior high school in a suburban area with 7th and 8th graders: 34 students; junior high school in a suburban area with 8th graders: 40 students).

### 3.2. Concept Maps and Ratings

#### 3.2.1. Students

**Figure A1:**
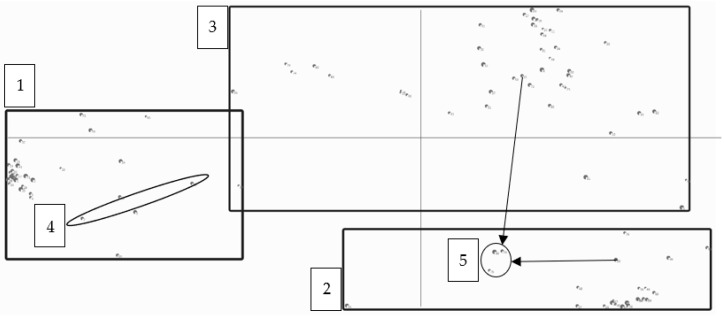
Concept map of girls (*n* = 30). Cluster 1: “bicycle and related equipment.” Cluster 2: “way to school.” Cluster 3: “requirements.” Cluster 4: “cycle training.” Cluster 5: “social behavior in road traffic.” A square indicates an original cluster, and a circle indicates a newly created cluster. An arrow indicates reallocation of an answer (illustrated as a dot) into another cluster.

The concept map of the 30 girls who completed the clustering task included the following five clusters, illustrating their needs to cycle to school daily (see Figure A1): (1) “Bicycle and related equipment” (30 answers), e.g., lock, bicycle, pump, helmet, bell, reflectors, lights, bicycle basket, repair kit; (2) “Way to school” (20 answers), e.g., less traffic and roadworks, crossing guards, (wide, signposted, extra) cycle paths, (direct, shorter, simple, even) route; (3) “Requirements” (41 answers), e.g., health and environmental awareness, fun, motivation and energy, breathing fresh air, good weather conditions (no rain, warm temperatures), saving time, company of friends or classmates, later start of school lessons, liking own bicycle; (4) “Cycle training” (2 answers), e.g., cycling test, ensure cycling abilities; (5) “Social behavior in road traffic” (5 answers), e.g., more mutual respect, friendly car drivers, paying attention to avoid accidents or dangerous situations.

**Figure A2:**
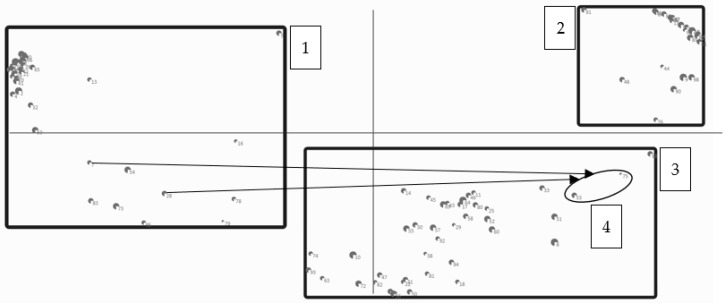
Concept map of boys (*n* = 53). Cluster 1: “bicycle and related equipment.” Cluster 2: “way to school.” Cluster 3: “requirements.” Cluster 4: “cycle training.” A square indicates an original cluster, and a circle indicates a newly created cluster. An arrow indicates reallocation of an answer (illustrated as a dot) into another cluster.

The concept map of the 53 boys who completed the clustering task included the following four clusters, illustrating their needs to cycle to school daily (see Figure A2): (1) “Bicycle and related equipment” (33 answers), e.g., lock, bicycle, pump, helmet, bell, reflectors, lights, bicycle basket, repair kit; (2) “Way to school” (23 answers), e.g., less traffic and roadworks, crossing guards, (wide, signposted, extra) cycle paths, (direct, shorter, simple, even) route; (3) “Requirements” (38 answers), e.g., health and environmental awareness, fun, motivation and energy, breathing fresh air, good weather conditions (no rain, warm temperatures), saving time, company of friends or classmates, later start of school lessons, liking own bicycle; (4) “Cycle training” (4 answers), e.g., cycling test, ensure cycling abilities, paying attention to avoid accidents or dangerous situations.

All four and five clusters identified in boys or girls, respectively, were rated as either unimportant/neutral or unfeasible/neutral on the Likert scale (see Table 1).

#### 3.2.2. Mothers

**Figure A3:**
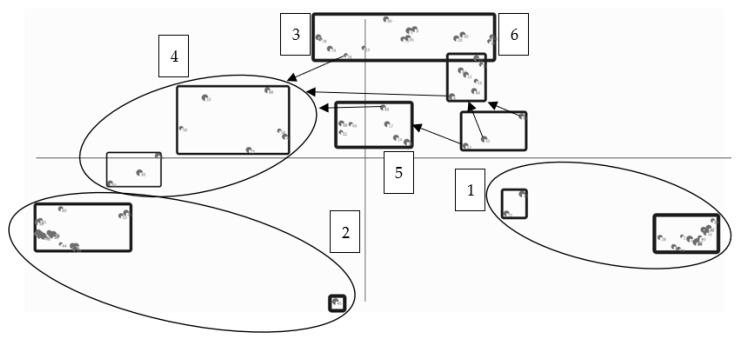
Concept map of mothers (*n* = 9). Cluster 1: “bicycle and related equipment.” Cluster 2: “way to school.” Cluster 3: “requirements.” Cluster 4: “motivation and social aspects.” Cluster 5: “role of the school.” Cluster 6: “role of parents.” A square indicates an original cluster, and a circle indicates a newly created cluster. An arrow indicates reallocation of an answer (illustrated as a dot) into another cluster.

The concept map of the nine mothers who completed the clustering task included the following six clusters, illustrating their perceptions of what children and adolescents need to cycle to school daily (see Figure A3): (1) “Bicycle and related equipment” (26 answers), e.g., lock, (cool) bicycle, (cool) helmet, reflectors, (cool) signal clothing, (strip) lights, carrier systems, bicycle basket; (2) “Way to school” (24 answers), e.g., road lighting, (wide) cycle paths, less traffic, (uncomplicated, interesting, optimal) route, no large roadworks, crossing guards, combination of active and passive parts, speed limit; (3) “Requirements” (13 answers), e.g., health (awareness), sense of safety, self-confidence, knowledge of traffic rules, orientation skills, outdoor affinity, fitness, liking to cycle, cycling experiences; (4) “Motivation and social aspects” (12 answers), e.g., company of friends, classmates, or siblings (group trips with meeting points), sense of community, breathing fresh air; (5) “Role of the school” (7 answers), e.g., storage facilities, no vandalism, cycling projects, lighter schoolbag; (6) “Role of parents” (8 answers), e.g., trust, not taking the child to school by car, role models (obligatory helmet wearing, outdoor affinity).

All six clusters identified in mothers were rated as either unimportant/neutral or unfeasible/neutral/feasible on the Likert scale (see Table 2).

#### 3.2.3. Teachers

**Figure A4:**
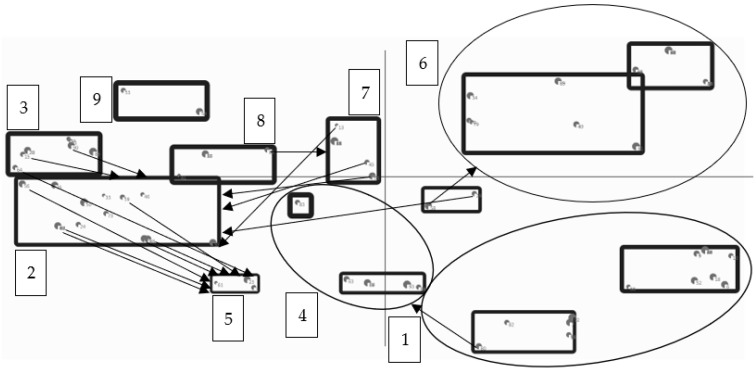
Concept map of female teachers (*n* = 5). Cluster 1: “bicycle and related equipment.” Cluster 2: “motivation and social aspects.” Cluster 3: “awareness.” Cluster 4 “financial aspects.” Cluster 5: “information and services.” Cluster 6: “way to school.” Cluster 7: “storage and changing room.” Cluster 8: “role of parents.” Cluster 9: ”sense of safety.” A square indicates an original cluster, and a circle indicates a newly created cluster. An arrow indicates reallocation of an answer (illustrated as a dot) into another cluster.

The concept map of the five female teachers who completed the clustering task included the following nine clusters, illustrating their perceptions of what children and adolescents need to cycle to school daily (see Figure A4): (1) “Bicycle and related equipment” (20 answers), e.g., lock, (cool) bicycle, (cool) helmet, reflectors, pump, lights; (2) “Motivation and social aspects” (15 answers), e.g., fun, incentives (scoring system, tests, class contests), rewards (certificate, price), sense of community, positive experiences in road traffic, sport interest, role models (friends, parents, teachers, siblings, classmates), good weather conditions (no rain, warm temperatures); (3) “Awareness” (5 answers), e.g., health and environmental awareness, cycling is cool (trendsetting), seeing the bicycle as sport object and means of transportation; (4) “Financial aspects” (9 answers), e.g., financial support to buy a bicycle and related equipment, appropriate clothing or a bicycle pool for cycle trainings at school; (5) “Information and services” (10 answers), e.g., information about appropriate clothing (rain jacket, pants) and carrier systems, repair service and bicycle flea market at school, information evening on advantages (environment and climate, health and fitness, saving money for fuel and public transport tickets, mobility, and independence), cycle training including traffic rules, kick-off event, school projects; (6) “Way to school” (21 answers), e.g., road lighting, cycle paths, orientation skills, less traffic around the school, cycle path guide, nice route, group trips with meeting points for friends, speed limit; (7) “Storage and changing room” (8 answers), e.g., (roofed, monitored) bicycle rack, access to changing rooms; (8) “Role of parents” (4 answers), e.g., not taking the child to school by car, support, traffic education, confidence in child; (9) “Sense of safety” (2 answers), i.e., everyone can cycle to school.

**Figure A5:**
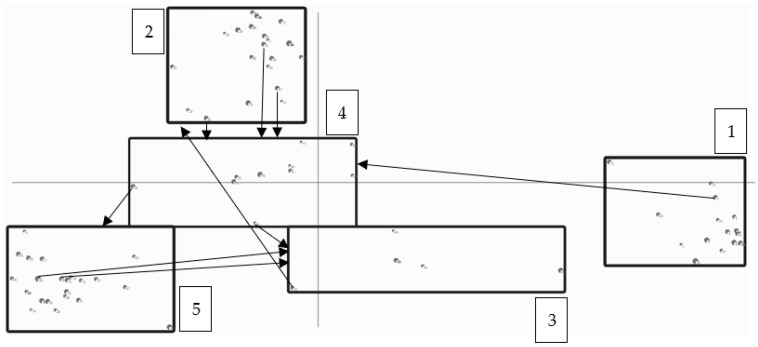
Concept map of male teachers (*n* = 5). Cluster 1: “bicycle and related equipment.” Cluster 2: “motivation, social aspects and awareness.” Cluster 3: “financial aspects.” Cluster 4: “information and services.” Cluster 5: “infrastructure.” A square indicates an original cluster. An arrow indicates reallocation of an answer (illustrated as a dot) into another cluster.

The concept map of the five male teachers who completed the clustering task included the following five clusters, illustrating their perceptions of what children and adolescents need to cycle to school daily (see Figure A5): (1) “Bicycle and related equipment” (20 answers), e.g., lock, (cool) bicycle, (cool) helmet, reflectors, pump, lights; (2) “Motivation, social aspects and awareness” (27 answers), e.g., parents not taking the child to school by car, role models (friends, parents, teachers, siblings, classmates), health and environmental awareness, cycling is cool (trendsetting), incentives (scoring system, class contests), fun, parental support, group trips with meeting points for friends, rewards (certificate, price), sense of community, positive experiences in road traffic, giving the feeling that everyone can cycle, parental confidence in child, sport interest, seeing the bicycle as sport object and means of transportation, good weather conditions (no rain, warm temperatures), saving time; (3) “Financial aspects” (10 answers), e.g., financial support to buy a bicycle and related equipment, appropriate clothing or a bicycle pool for cycle trainings at school; (4) “Information and services” (12 answers), e.g., information about appropriate clothing (rain jacket, pants) and carrier systems, repair service and bicycle flea market at school, traffic education (cycle training including traffic rules and test), information evening on advantages (environment and climate, health and fitness, saving money for fuel and public transport tickets, mobility and independence), kick-off event, school projects; (5) “Infrastructure” (25 answers) including the “way to school” and “storage and changing room,” e.g., (roofed, monitored) bicycle rack, road lighting, cycle paths, speed limit, less traffic around the school, cycle path guide, access to changing rooms, nice route.

All five and nine clusters identified in male or female teachers, respectively, were rated as either neutral/important or unfeasible/neutral/feasible on the Likert scale (see Table 3).

## 4. Discussion

Our concept mapping study analyzed factors needed by children and adolescents aged 12 to 15 in Germany to ride their bicycles to school every day based on gender perspectives of students, parents, and teachers. We found that every boy but not every girl owned a bicycle; this should be considered in future interventions (e.g., provision of bicycles) because only students who own a bicycle can actually cycle to school. Additionally, considerably more boys than girls cycled to school, in line with previous research in Germany [11]. Despite asking a similar question in this study, however, cycling rates were much higher (girls: 44.4% vs. 20.6%, boys: 72.9% vs. 23.8%) [11], suggesting that rates of cycling to school might have changed from 2003–2006 [11] and 2019. Nevertheless, cycling to school was not a daily habit in our sample, indicating room for improvement. Even though our low ICC, calculated for the within and between variance of days per week students cycled to the three participating schools, is in line with previously reported ICCs for group-randomized intervention designs (0.1 to 0.3 [32]), very low ICCs of 0.05 or 0.01 can lead to a meaningful bias in the results of significance tests [30] due to variances. Following this, researchers should keep a potential variance in mind when planning a school-based bicycle intervention (i.e., several schools per intervention condition in group-randomized designs). Contrary to our intention, we could not analyze fathers’ perspectives and compare them with mothers’ data due to the small number of complete data for fathers. Between girls and boys, we found one difference in clustering. Only girls clustered answers into “social behavior in road traffic.” For each cluster, ratings of importance and feasibility were very similar in girls and boys. Between female and male teachers, we found differences in four clusters. Male teachers classified clusters into broader subjects, i.e., the cluster “motivation and social aspects” included “awareness” and the cluster “infrastructure” included “way to school” as well as “storage and changing room.” Only female teachers clustered answers into “role of parents” and “sense of safety.” For each cluster, ratings of importance and feasibility were very similar in female and male teachers.

### 4.1. Clusters in Concept Maps

#### 4.1.1. Similar Clusters in Concept Maps of Mothers and Students and Teachers Independent of Gender

The need for a “bicycle and related equipment” (e.g., lock, bicycle, helmet, reflectors, lights) was stated by students, teachers, and mothers. When children and adolescents want to cycle to school, the basic necessity of bicycle ownership is indisputable. As every boy and nearly every girl in our sample owned a bicycle, providing all students in our sample with a bicycle in a future intervention does not seem necessary. Regarding bicycle-related equipment, previous research remained unclear on whether “the equipment of a child’s bicycle is a potential determinant of cycling to school” [33] (p. 290). Nevertheless, the only overall effective bicycle intervention in our recent systematic review [12] was conducted in the USA and provided every child with a bicycle and related equipment (i.e., helmet, lock, lights) prior to the beginning of the intervention [34,35]. In our study, girls rated a lock and brakes as important equipment, whereas boys rated only a lock as important equipment. According to German Road Traffic Licensing Regulations, researchers might provide specific equipment (i.e., a bell, two independent brakes, two anti-slip and screwed-on pedals with two yellow reflectors shining to the front and rear, white front and red rear light, two reflectors per wheel, white front, and a red rear reflector [36]) to ensure the roadworthiness and safety of bicycles in an intervention.

Factors related to the “way to school,” such as less traffic (especially around the school), (wide, signposted, extra) cycle paths, a cycle path guide, and an even route, were identified across all concept maps of students, teachers, and mothers. Traffic density and type of cycle paths (e.g., evenness) were reported as the most important factors for a cycling-friendly environment for children in previous research [37]. In addition, a cycle path guide (e.g., parental accompaniment while cycling) was positively associated with cycling behavior in children [38]. Comprehensive changes related to the way to school in school-based interventions require the involvement of municipal stakeholders.

Personal needs were represented in the cluster of students as “requirements” (e.g., motivation, company of friends or classmates), in the clusters of teachers as “motivation and social aspects” and “awareness,” and in clusters of mothers as “requirements” and “motivation and social aspects.” Because previous research also underlined the role of personal factors [39], it might be relevant to address the three basic psychological needs “autonomy, competence, and relatedness” of Self-Determination Theory [40] in future interventions with children and adolescents for long-term internalization of cycling-to-school behavior.

#### 4.1.2. Unique Clusters in Concept Maps of Students (In) Dependent of Gender

“Cycle training” (e.g., cycling test, ensure cycling abilities) was identified by both girls and boys. To overcome barriers to cycle to school, cycle training is recommended by the “NZ Transport Agency” [41]. However, results from a previous study demonstrated that providing only cycle training on the school playground during physical education lessons was not effective in children’s cycling-to-school behavior [42]. Following this, cycle training content should not only be chosen carefully based on needs mentioned by students but should also be implemented in the natural environment in future interventions to promote cycling to school.

The cluster “social behavior in road traffic” (e.g., more mutual respect, friendly car drivers, paying attention to avoid accidents or dangerous situations) was mentioned only by girls. Besides theoretical knowledge of traffic rules and practical cycling skills, social competences are considered essential for responsible and anticipated participation in road traffic by the “Standing Conference of the Ministers of Education and Cultural Affairs” (KMK) in Germany [43]. To acquire these competences, the KMK assigns mobility and traffic education to schools [43]. The reason boys did not mention this cluster might be explained by the observation in Germany that boys have a higher risk of injury in road traffic (accidents) due to more risky behavior than girls [44]. Therefore, the topic “social behavior in road traffic” is an important element in mobility and traffic education (especially for boys to reflect on the impact of their gender role) [44].

#### 4.1.3. Similar and Unique Clusters in Concept Maps of Mothers, and Teachers (In) Dependent of Gender

Mothers and female teachers mentioned the “role of parents,” e.g., not taking the child to school by car. Several theoretical models, for example, the M-CAT [14] or the “Social-ecological model of the correlates of active transportation” [45], consider parents’ role as supporters or decision-makers. However, this role’s impact decreases as the child matures [14]. Additionally, the 12- to 15-year-olds in our sample did not acknowledge their parents’ role. Therefore, future interventions for this age group should empower parents to support children’s need for autonomy, independence, and personal responsibility regarding mobility.

In line with theoretical models [14,45], mothers mentioned the cluster “role of the school” (e.g., storage facilities, no vandalism, cycling projects, lighter schoolbags). Additionally, the KMK has defined the teaching and educational role of mobility and traffic in schools [43], but neither students nor teachers acknowledged this. Therefore, the role of schools should be emphasized in future school-based bicycle interventions.

Mentioned by both female and male teachers, “storage and changing room” referred, for example, to a roofed and monitored bicycle rack or access to changing rooms. Even though students and mothers did not identify this cluster, the lack of or poor quality changing rooms and bicycle racks in schools have been previously reported to influence children and adolescents’ PA behavior negatively [19]. Students and mothers might not have identified this need if they were satisfied by conditions at their school, but it might be relevant at schools with poor conditions.

Independent of gender, teachers identified the need for “financial aspects” (e.g., financial support to buy a bicycle and related equipment, appropriate clothing, or a bicycle pool for cycle trainings at school). In line with this, M-CAT states parents’ income as a relevant factor for ACTS [14]. However, mothers and students did not mention this cluster, so financial aspects might not be a major issue for parents (who bear financial responsibility) or for students. Our assumption might be reflected in students’ pervasive bicycle ownership because in our study sample, every boy owned a bicycle and only 12.2% of girls did not. This also makes it unnecessary to provide an entire bicycle pool for cycle training at the three participating schools.

Independent of gender, teachers identified the need for “information and services,” e.g., information about appropriate clothing (rain jacket, pants) and carrier systems, repair service and a bicycle flea market at school, an information evening on advantages (environment and climate, health and fitness, saving money for fuel and public transport tickets, mobility and independence), cycle training including traffic rules, a kick-off event, and school projects (bicycle tour, project day). In grades 5 to 10 (students aged 10 to 15), the KMK explicitly mentions the provision of informational manuals and materials (e.g., about environment and climate), implementation of activities (e.g., ecological school trips), and cooperation with out-of-school partners (e.g., bicycle repair shops) to promote students’ independent mobility [43]. However, provision of information and services might be feasible but not crucial in the development of future school-based bicycle interventions. Perhaps this is why students and mothers did not consider this need relevant.

Clusters between female and male teachers differed as only female teachers clustered answers into “sense of safety,” i.e., giving the feeling that everyone can use a bicycle to engage in ACTS. As an important barrier to ACTS, children’s personal safety fears were also identified in previous research [39], and this cluster might be reflected in students’ identified needs for cycle training and social behavior in road traffic. Thus, future school-based bicycle interventions should attempt to establish feelings of safety among students.

### 4.2. Importance and Feasability

Across students, mothers, and teachers, Likert scale ratings of the degree of importance and feasibility of their provided answers showed not a single extreme response, i.e., very (un) important or (un) feasible. Participants noticeably tended to choose the unimportant/unfeasible or neutral rating categories so that ratings were very similar. Undecidedness [46], lack of motivation [47] due to the large number of participants’ answers (students: 98; parents: 90; teachers: 94) that had to be rated, or a question not specific enough [48] might have led to this central tendency bias. Therefore, findings on ratings should be interpreted with caution.

### 4.3. Strengths and Limitations

Quantitative analysis of qualitative data in the concept mapping approach could be seen as a strength of this study. Additionally, stratified gender analyses provide a deeper understanding of different perspectives on what is needed for cycling to school. We found one and two unique gender-dependent cluster(s) in students and teachers, respectively. A limitation might be that group sessions were not conducted separately for females and males. Moreover, we could not include grades 7 and 8 in each session at every school since the schools decided the participating grades. Contrary to our previous intention, teachers did not allow us to divide classes of 22 to 32 into smaller groups of 8 to 10 students. This made conducting sessions challenging in terms of personnel, time, and resources (e.g., sufficient computers, stable internet connections) but led to a higher student recruitment rate (i.e., planned: 48; recruited: 136), which is a major strength. In general, participants were interested in the concept mapping sessions and liked getting involved by providing their opinions, which gave us an insight into their perceptions. This might explain why we also exceeded our recruitment goals for parents and teachers (58 and 29 instead of 25 each). Interestingly, more mothers than fathers contributed to the concept mapping sessions. This gender bias in our online survey’s response rate aligns with previous research [49] and might be explained by differences in perceived parenting responsibilities. Due to small sample sizes as well as high drop-out rates of parents and teachers and to the few regions sampled in Germany, our findings cannot be generalized and might differ in comparison with other nations. Nevertheless, studies using the concept mapping approach in very small samples of five to eight participants are not unusual [50].

Throughout our sessions, we were confronted with several difficulties. Participants complained about the time-consuming involvement (e.g., too many answers), the type of survey (i.e., paper/pencil) and other participants’ “absurd” answers (e.g., “I need training wheels”). Furthermore, non-native speakers (e.g., refugees) struggled especially with the amount of information in German. Generally, participants also found it difficult to separate ratings between importance and feasibility. In addition, participants struggled with rating tasks when answers were not applicable to their situations (e.g., students, whose parents were not worried, struggled how to rate “reduction of fear in parents”). Due to technical failures that occurred throughout the sessions with both online programs (Survalyzer, Ariadne), we could not ensure completeness of data (an inclusion criterion for Ariadne analyses). Some of these difficulties might have led to a lowered willingness and motivation to participate, thus possibly explaining the central tendency bias in importance and feasibility ratings and the relatively high drop-out rates, particularly in parents (79.3%) and teachers (62.1%), in contrast with students (26.5%).

### 4.4. Recommendations

Based on our experience from this study, we recommend modifying the concept mapping approach for such a complex subject and/or for its application in large groups due to school rules. To achieve participants’ maximum commitment and to reduce their burden, we suggest conducting all sessions online (especially the clustering task), but in school groups supervised by researchers to ensure personal contact. Another advantage of online sessions is the immediate digital availability of collected data, which eliminates the risk of errors in transferring data manually. We further recommend removing the second online session in which participants check the clarity of answers. Instead, the first online session could be completed with a group brainstorming phase including a clarity check and a removal of duplicates. To make “ACTS by bicycle” less complex for participants (i.e., fewer answers), the main question in the first session could be specified according to factors in the “Social-ecological model of the correlates of active transportation” [45] (e.g., the needs in terms of environmental factors only). Still, to acquire a comprehensive picture of needs, the concept mapping approach could be conducted for each factor of this model based on more specific questions in different samples (e.g., different classes) in the same schools. Another possibility could be to restrict the number of answers to a more manageable number (e.g., 40 to 70) [51] by checking duplicates more strictly and combining answers after session one.

To maintain participants’ motivation and to address their need for time efficiency, each provided answer could be immediately rated for importance and feasibility. In the second study session, we changed this when students received the paper/pencil version and reflected positive experiences with the procedure. Future studies could optimize rating tasks to avoid central tendency bias and inconclusive findings by replacing the five-point Likert scale with an even-point scale, i.e., a scale without a midpoint, which forces participants to choose positively or negatively. Independent of language skills, the majority of students had problems answering the question about frequency of “cycling to school (days/week)” because they cycled every day in the summer but took the bus or train in the winter. These seasonal differences align with previous Norwegian findings that reported large variations in fall (52%), winter (3%), spring (51%) [52], and in summer (22%) compared to winter (12%) [53]. Therefore, we highly recommend modifying this question to consider potential seasonal variations in surveys and to take different weather conditions into account when developing an intervention. Finally, the program Ariadne appeared to be prone to error and was perceived to be user-unfriendly, so we recommend that this program be improved for future concept mapping studies.

## 5. Conclusions

This study provides insight into the perceptions of girls and boys, mothers, and female and male teachers on what 12- to 15-year-old children and adolescents living in Germany need to cycle daily to school. Between genders, we found more overall similarities than differences in clusters. Students and teachers, independent of gender, and mothers mentioned the need for “bicycle and related equipment,” “way to school,” and “personal factors.” Additionally, independent of gender, students identified “cycle training” and teachers a “storage and changing room,” “financial aspects,” and “information and services” as children and adolescents’ needs. Furthermore, girls identified the need for “social behavior in road traffic,” mothers and female teachers the “role of parents,” and female teachers the “sense of safety.” However, boys and male teachers did not mention these three needs. Only mothers clustered the “role of the school.” Furthermore, we found bias in clusters’ importance and feasibility ratings and could not draw final conclusions. Nevertheless, we hope that the combined perceptions complement each other to support the uptake and long-term maintenance of ACTS by bicycle. Our findings can be used to inform students, mothers, and teachers about their mutual perceptions and can help researchers develop school-based interventions to promote daily cycling to school.

## Figures and Tables

**Table 1:** Students’ clusters and ratings of importance and feasibility by gender.

Name of Cluster	Rating of Importance		Rating of Feasibility	
	Girls (*n* = 34)	Boys (*n* = 59)	Girls (*n* = 32)	Boys (*n* = 50)
Bicycle and related equipment	3.4 ± 1.2	3.1 ± 1.3	3.5 ± 1.2	3.5 ± 1.4
Way to school	3.1 ± 1.2	2.9 ± 1.2	2.9 ± 1.3	2.8 ± 1.3
Requirements	3.0 ± 1.2	3.0 ± 1.4	2.9 ± 1.3	3.0 ± 1.4
Cycle training	3.6 ± 1.2	3.4 ± 1.3	3.7 ± 1.2	3.5 ± 1.4
Social behavior in road traffic	3.3 ± 1.2	-	3.1 ± 1.3	-

Means ± standard deviation.

**Table 2:** Mothers’ clusters and ratings of importance and feasibility (*n* = 9).

Name of Cluster	Rating of Importance	Rating of Feasibility
Bicycle and related equipment	3.5 ± 1.0	4.3 ± 0.7
Way to school	3.1 ± 1.0	2.9 ± 0.9
Requirements	3.5 ± 1.0	3.8 ± 0.7
Motivation and social aspects	2.5 ± 1.0	2.9 ± 0.9
Role of the school	3.5 ± 0.9	3.6 ± 0.9
Role of parents	2.9 ± 1.0	3.7 ± 0.9

Means ± standard deviation.

**Table 3:** Teachers’ clusters and ratings of importance and feasibility by gender.

Name of Cluster	Rating of Importance		Rating of Feasibility	
	Female Teachers (*n* = 5)	Male Teachers (*n* = 5)	Female Teachers (*n* = 5)	Male Teachers (*n* = 5)
Bicycle and related equipment	3.6 ± 0.8	3.7 ± 0.7	3.5 ± 0.7	3.4 ± 0.7
Motivation and social aspects	3.6 ± 1.0	3.6 ± 0.8	3.6 ± 0.9	3.5 ± 0.6
Awareness	4.0 ± 0.9		4.0 ± 0.8	
Financial aspects	3.3 ± 0.9	3.3 ± 1.0	2.8 ± 0.9	2.8 ± 1.1
Information and services	3.2 ± 1.0	3.4 ± 0.7	4.2 ± 0.8	3.7 ± 0.7
Way to school	3.6 ± 0.9	3.6 ± 0.9	3.3 ± 0.7	3.2 ± 1.0
Storage and changing room	3.3 ± 1.2		3.6 ± 1.0	
Role of parents	4.1 ± 0.7	-	3.6 ± 0.8	-
Sense of safety	4.3 ± 0.7	-	4.0 ± 0.4	-

Means ± standard deviation.

## Appendix A

**Table A1:** Overview of sections, questions, and response options of the first concept mapping session with students.

Section	Questions	Response Option(s)
Personal characteristics	age (years)	open-end
	gender	(a) female (b) male (c) diverse
	school’s region	(a) urban (b) suburban
	school’s zip-code	open-end
	educational level	(a) high school (b) junior high school
	class level	(a) 7 (b) 8
	bicycle ownership	(a) yes (b) no
	ability to cycle	(a) yes (b) no
	cycling to school	(a) yes (b) no
	cycling to school (days/week)	0–5
	shortest cycling distance home/school (km ^1^)	open-end
Warm-up question	Why do or don’t you cycle to school?	open-end
Main question	What do you need to cycle to and from school on a daily basis?	open-end

^1^ km = kilometer.

**Table A2:** Personal characteristics of participating students by gender.

Personal Characteristics	Female (*n* = 51)	Male (*n* = 83)	*p*-Value ^3^	Diverse (*n* = 2)	Response Rate (N = 136)
Age (years in M ± SD ^1^)	13.1 ± 0.9	13.1 ± 0.9	0.778	13.0 ± 0.000	123
Educational level (school’s region)			0.156		136
(a) high school (urban)	13 (25.5%)	31 (37.3%)		0 (0%)	
(b) junior high school (suburban)	38 (74.5%)	52 (62.7%)		2 (100%)	
Class level			0.267		136
(a) 7th grade	22 (43.1%)	44 (53.0%)		0 (0%)	
(b) 8th grade	29 (56.9%)	39 (47.0%)		2 (100%)	
Bicycle ownership			0.004 **		123
(a) yes	43 (87.8%)	72 (100%)		2 (100%)	
(b) no	6 (12.2%)	0 (0%)		0 (0%)	
Ability to cycle			n.a. ^4^		123
(a) yes	49 (100%)	72 (100%)		2 (100%)	
(b) no	0 (0%)	0 (0%)		0 (0%)	
Cycling to school			0.002 **		117
(a) yes	20 (44.4%)	51 (72.9%)		1 (50.0%)	
(b) no	25 (55.6%)	19 (27.1%)		1 (50.0%)	
Cycling to school (days/week in M ± SD ^1^)	1.6 ± 2.0	2.7 ± 2.0	0.003 **	1.5 ± 2.1	116
Shortest cycling distance home/school (km ^2^ in M ± SD ^1^)	3.3 ± 2.6	4.0 ± 3.1	0.307	8.0 ± 9.9	122

^1^ means ± standard deviation, ^2^ km = kilometer, ^3^ *p*-values were calculated for gender differences (female vs. male) using U-test or Chi-squared tests, ^4^ n.a. = not applicable, ** = 0.01 ≥ *p* > 0.001.

**Table A3:** Overview of sections, questions, and response options of the first concept mapping session with parents.

Section	Question(s)	Response Option(s)
Personal characteristics	age (years)	open-end
	gender	(a) female (b) male (c) diverse
	age of child (years)	(a) 12 (b) 13 (c) 14 (d) other
	gender of child	(a) daughter (b) son
	child’s school region	(a) urban (b) suburban
	child’s school zip-code of child	open-end
	educational level of child	(a) high school (b) junior high school
	class level of child	(a) 7 (b) 8
	bicycle ownership of child	(a) yes (b) no
	child’s ability to cycle	(a) yes (b) no
	cycling to school of child	(a) yes (b) no
	cycling to school of child (days/week)	0–5
	shortest cycling distance home/school of child (km ^1^)	open-end
	bicycle ownership	(a) yes (b) no
	ability to cycle	(a) yes (b) no
	work (days/week)	0–5
	cycling to work	(a) yes (b) no
	cycling to work (days/week)	0–5
	shortest cycling distance home/work (km ^1^)	open-end
Warm-up question	Why does or doesn’t your child cycle to school?	open-end
Main question	What does your child need to cycle to and from school daily?	open-end

^1^ km = kilometer.

**Table A4:** Overview of sections, questions, and response options of the first concept mapping session with teachers.

Section	Question(s)	Response Option(s)
Personal characteristics	age (years)	open-end
	gender	(a) female (b) male (c) diverse
	work experience (years)	open-end
	school’s region	(a) urban (b) suburban
	school’s zip-code	open-end
	educational level	(a) high school (b) junior high school
	class level of target group	open-end
	cycling to school of target group (%)	open-end
	cycling to school of target group (days/week)	0–5
	bicycle ownership	(a) yes (b) no
	ability to cycle	(a) yes (b) no
	work (days/week)	0–5
	cycling to work	(a) yes (b) no
	cycling to work (days/week)	0–5
	shortest cycling distance home/work (km ^1^)	open-end
Warm-up question	Why do or don’t your students cycle to school?	open-end
Main question	What do your students need to cycle to and from school daily?	open-end

^1^ km = kilometer.

**Table A5:** Personal characteristics of participating parents by gender.

Personal Characteristics	Female (*n* = 35)	Male (*n* = 8)	*p*-Value ^3^	Response Rate
				(N = 43)
Age (years in M ± SD ^1^)	46.8 ± 5.1	52.1 ± 5.2	0.034 *	42
Age of child (years in M ± SD ^1^)	12.6 ± 0.7	13.0 ± 0.8	0.145	42
Gender of child			1	43
(a) daughter	(a) 12 (34.3%)	(a) 3 (37.5%)		
(b) son	(b) 23 (65.7%)	(b) 5 (62.5%)		
Educational level (school’s region) of child			1	43
(a) high school (urban)	(a) 15 (42.9%)	(a) 4 (50.0%)		
(b) junior high school (suburban)	(b) 20 (57.1%)	(b) 4 (50.0%)		
Class level of child			1	43
(a) 7th grade	(a) 23 (65.7%)	(a) 6 (75.0%)		
(b) 8th grade	(b) 12 (34.3%)	(b) 2 (25.0%)		
Bicycle ownership of child			n.a. ^4^	42
(a) yes	(a) 34 (100%)	(a) 8 (100%)		
(b) no	(b) 0 (0%)	(b) 0 (0%)		
Child’s ability to cycle			n.a. ^4^	42
(a) yes	(a) 34 (100%)	(a) 8 (100%)		
(b) no	(b) 0 (0%)	(b) 0 (0%)		
Cycling to school of child			1	40
(a) yes	(a) 22 (66.7%)	(a) 5 (71.4%)		
(b) no	(b) 11 (33.3%)	(b) 2 (28.6%)		
Cycling to school of child (days/week in M ± SD ^1^)	2.6 ± 2.3	3.1 ± 2.2	0.985	40
Shortest cycling distance home/school of child (km ^2^ in M ± SD ^1^)	4.3 ± 3.2	5.2 ± 3.2	0.432	42
Bicycle ownership			1	42
(a) yes	(a) 33 (97.1%)	(a) 8 (100%)		
(b) no	(b) 1 (2.9%)	(b) 0 (0%)		
Ability to cycle			n.a. ^4^	42
(a) yes	(a) 34 (100%)	(a) 8 (100%)		
(b) no	(b) 0 (0%)	(b) 0 (0%)		
Work (days/week in M ± SD ^1^)	3.7 ± 1.5	4.9 ± 0.4	0.004 **	42
Cycling to work			0.698	38
(a) yes	(a) 12 (40.0%)	(a) 4 (50.0%)		
(b) no	(b) 18 (60.0%)	(b) 4 (50.0%)		
Cycling to work (days/week in M ± SD ^1^)	1.3 ± 1.9	1.8 ± 2.2	0.549	38
Shortest cycling distance home/work (km ^2^ in M ± SD ^1^)	13.0 ± 14.4	7.9 ± 5.5	0.676	39

^1^ means ± standard deviation, ^2^ km = kilometer, ^3^ *p*-values were calculated for gender differences (female vs. male) using U-test or Chi-squared tests, ^4^ n.a. = not applicable, * = 0.05 ≥ *p* > 0.01, ** = 0.01 ≥ *p* > 0.001.

**Table A6:** Personal characteristics of participating teachers by gender.

Personal Characteristics	Female (*n* = 14)	Male (*n* = 13)	*p*-Value ^4^	Response Rate (N = 27)
Age (years in M ± SD ^1^)	43.3 ± 11.5	35.3 ± 8.9	0.068	27
Work experience (years in M ± SD ^1^)	15.2 ± 12.0	7.2 ± 5.6	0.068	27
Educational level (school’s region)			0.12	27
(a) high school (urban)	(a) 3 (21.4%)	(a) 7 (53.8%)		
(b) junior high school (suburban)	(b) 11 (78.6%)	(b) 6 (46.2%)		
Class level of target group (min/max ^2^)	6–9	6–9	n.a. ^5^	27
Cycling to school of target group (% in M ± SD ^1^)	40.0%	20.0% ± 15.8%	0.277	5
Cycling to school of target group (days/week in M ± SD ^1^)	5.0	4.0 ± 0.8	0.264	5
Bicycle ownership			1	27
(a) yes	(a) 13 (92.9%)	(a) 13 (100%)		
(b) no	(b) 1 (7.1%)	(b) 0 (0%)		
Ability to cycle			n.a. ^5^	27
(a) yes	(a) 14 (100%)	(a) 13 (100%)		
(b) no	(b) 0 (0%)	(b) 0 (0%)		
Work (days/week in M ± SD ^1^)	4.1 ± 0.8	4.9 ± 0.4	0.008 **	27
Cycling to work			0.107	26
(a) yes	(a) 6 (46.2%)	(a) 10 (76.9%)		
(b) no	(b) 7 (53.8%)	(b) 3 (23.1%)		
Cycling to work (days/week in M ± SD ^1^)	1.6 ± 2.1	2.8 ± 2.2	0.127	26
Shortest cycling distance home/work (km ^3^ in M ± SD ^1^)	8.9 ± 7.8	13.0 ± 12.9	0.382	27

^1^ means ± standard deviation, ^2^ min/max = minimum/maximum, ^3^ km = kilometer, ^4^ *p*-values were calculated for gender differences (female vs. male) using U-test or Chi-squared tests, ^5^ n.a. = not applicable, ** = 0.01 ≥ *p* > 0.001.
